# Effects of pH Values and H_2_O_2_ Concentrations on the Chemical Enhanced Shear Dilatancy Polishing of Tungsten

**DOI:** 10.3390/mi13050762

**Published:** 2022-05-12

**Authors:** Liang Xu, Lin Wang, Hongyu Chen, Xu Wang, Fangyuan Chen, Binghai Lyu, Wei Hang, Wenhong Zhao, Julong Yuan

**Affiliations:** 1 College of Mechanical Engineering, Zhejiang University of Technology, Hangzhou 310023, China; 2111902045@zjut.edu.cn (L.X.); 2112102013@zjut.edu.cn (L.W.); wx382935877@163.com (X.W.); chenfyupmc@163.com (F.C.); icewater7812@126.com (B.L.); whang@zjut.edu.cn (W.H.); whzhao6666@163.com (W.Z.); jlyuan@zjut.edu.cn (J.Y.); 2 Key Laboratory of Special Purpose Equipment and Advanced Processing Technology, Ministry of Education and Zhejiang Province, Zhejiang University of Technology, Hangzhou 310023, China

**Keywords:** chemical enhanced shear dilatancy, tungsten, polishing, surface quality

## Abstract

In order to obtain tungsten with great surface qualities and high polishing efficiency, a novel method of chemical enhanced shear dilatancy polishing (C-SDP) was proposed. The effects of pH values and H_2_O_2_ concentrations on the polishing performance of tungsten C-SDP were studied. In addition, the corrosion behaviors of tungsten in solutions with different pH values and H_2_O_2_ concentrations were analyzed by electrochemical experiments, and the valence states of elements on the tungsten surface were analyzed by XPS. The results showed that both pH values and H_2_O_2_ concentrations had significant effects on tungsten C-SDP. With the pH values increasing from 7 to 12, the *MRR* increased from 6.69 µm/h to 13.67 µm/h. The optimal surface quality was obtained at pH = 9, the surface roughness (*R*_a_) reached 2.35 nm, and the corresponding *MRR* was 9.71 µm/h. The *MRR* increased from 9.71 µm/h to 34.95 µm/h with the H_2_O_2_ concentrations increasing from 0 to 2 vol.%. When the concentration of H_2_O_2_ was 1 vol.%, the *R*_a_ of tungsten reached the lowest value, which was 1.87 nm, and the *MRR* was 26.46 µm/h. This reveals that C-SDP technology is a novel ultra-precision machining method that can achieve great surface qualities and polishing efficiency of tungsten.

## 1. Introduction

Due to the advantages of high melting point, great corrosion resistance, superior electrical conductivity, and high-temperature strength [1,2,3], tungsten is widely used in integrated circuits, nuclear material, the military industry, medical equipment, and other fields [4,5,6]. The surface quality of the material has a significant influence on the service performance and service life of the workpiece. For example, as the primary candidate for plasma facing materials (PFMs) in the diverter of the ITER and DEMO fusion reactors, tungsten needs to withstand the impact of high-energy particles. The surface quality will affect its radiation resistance to a certain extent, thus affecting the service life of the nuclear fusion reactor [7]. In semiconductor devices, tungsten is deposited on the device surface by chemical vapor deposition. The surface quality of tungsten film will affect the interconnect performance of devices, and therefore the surface of tungsten film needs to be polished [8]. Ultra-precision polishing is the final processing method to reduce the surface roughness of workpiece, remove the damaged layer, and obtain high surface accuracy and excellent surface quality [9,10,11]. However, as a typical hard and brittle material, there are great challenges in the ultra-precision polishing of tungsten due to the high hardness, high brittleness, and great wear resistance of material [12,13].

In order to achieve high-efficiency and high-quality polishing, researchers have conducted extensive research on tungsten polishing methods and processes. Poddar et al. [14] used a mixed oxidant composed of H_2_O_2_ and Fe(NO_3_)_3_ for chemical–mechanical polishing of tungsten and found that the mixed oxidant would generate OH with stronger oxidizing ability, and its removal efficiency was higher than that of a single oxidant. Lim et al. [15] found that the polishing rate could be divided into two regions with increases in Fe(NO_3_)_3_ concentration; when Fe(NO_3_)_3_ concentration was less than 0.1 wt.%, the polishing rate of tungsten increased rapidly, while when the Fe(NO_3_)_3_ concentration was higher than 0.1 wt.%, the polishing rate increased slowly. Han et al. [16] used an alkaline electrolyte containing 0.27 mol/L NaOH for electrochemical polishing of tungsten under the optimal electrode gap width, and surface roughness (*R*_a_) of the polished tungsten reached 7.5 nm. Based on polishing experiments and theoretical analysis, Wang et al. [17] proposed that electrochemical polishing of tungsten should be divided into three stages: corrosion stage, bright stage, and pitting stage. Under the optimal process, surface roughness *S*_a_ was only 3.73 nm after polishing for 10 min. Chen et al. [18] proposed a high-efficiency electrochemical polishing method for tungsten surfaces combining forced convection and natural convection. After electrochemical polishing by forced convection for 3 min and natural convection for 3 min, surface roughness *R*_a_ was reduced to 17.2 nm. Zhou et al. [19] developed a dynamic electrochemical polishing process using a bi-layer NaOH electrolyte to uniformly polish tungsten microfluidic molds. With the optimized parameters, surface roughness *S*_a_ was reduced from 205.98 nm to 4.14 nm after 10 cycles of dynamic electrochemical polishing. Tungsten will be widely used in various fields in the future because of its excellent comprehensive mechanical properties. Therefore, it is particularly important to explore new ultra-precision polishing methods to improve the surface quality and service life of tungsten workpieces.

Shear Dilatancy Polishing (SDP) is a high-efficiency, high-quality, low-cost surface polishing method that has emerged in recent years. The principle is to use viscoelastic material with non-Newtonian fluid properties to prepare a specific shear pad in order to enhance the control of abrasive particles and improve the stress evenness and contact pressure on the workpiece surface based on the shear dilatancy and solidification effects under high-pressure and high-speed conditions to finally achieve high-efficiency and high-quality polishing. The scratching effect of abrasives on the workpiece is the key to achieving material removal [20,21]. Compared with conventional polishing pads, the dilatancy pad can hold more abrasives. Doi et al. [22,23] used viscoelastic materials such as asphalt and potato starch to prepare a specific dilatancy pad, which could reduce the surface defects caused by stress concentrations in local areas during processing. Results showed that under low–medium speed/pressure, the material removal rate of the SiC wafer after the dilatancy pad processing was more than three times of that after metal tin plate processing, the surface scratches after the dilatancy pad processing were lower than 1% of the latter, and the depth of the subsurface damage layer was less than 10% of the latter.

In this study, Chemical enhanced Shear Dilatancy Polishing (C-SDP) as a novel ultra-precision polishing method was proposed to obtain high surface quality tungsten. The effects of pH values and H_2_O_2_ concentrations of polishing slurry on material removal rate (*MRR*) and surface roughness (*R*_a_) in the tungsten C-SDP process were studied. In addition, the corrosion behaviors of tungsten in solutions with different pH values and H_2_O_2_ concentrations were analyzed by electrochemical experiments, and the valence states of elements on the tungsten surface were analyzed by XPS.

## 2. Principle of Chemical Enhanced Shear Dilatancy Polishing

**Figure 1:**
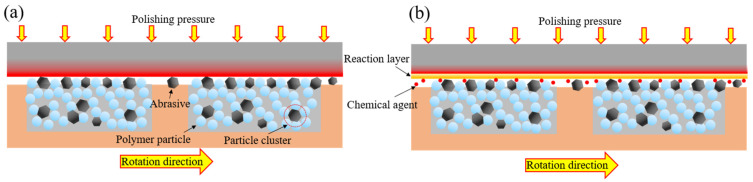
Schematic illustrations of the polishing principle: (**a**) Shear Dilatancy Polishing (SDP); (**b**) Chemical enhanced Shear Dilatancy Polishing (C-SDP).

Figure 1a,b are schematic illustrations of the SDP and C-SDP principles, respectively. In the SDP processing, the abrasives can be trapped in the viscoelastic material to avoid the height difference caused by different abrasive particle sizes and improve the uniformity of force on the workpiece surface. C-SDP is a polishing method with the synergistic effect of mechanical action and chemical action. It selectively removes workpiece surface roughness peaks based on chemical etching of oxidants and efficient mechanical removal of the dilatancy pad, resulting in higher efficiency and greater surface quality of workpieces than can be achieve using SDP.

During C-SDP processing, the chemical polishing slurry contacts the tungsten surface so that a chemical reaction occurs, forming a passivation film with a lower hardness than tungsten. Moreover, relative movement between the dilatancy pad and tungsten workpiece will happen. Under shear force and pressure, viscoelastic material with non-Newtonian fluid properties in the contact area instantly exhibits shear dilatancy and solidification effects, forming a “flexible fixed abrasive tool (FFAT)” in the processing area, which can realize micro-cutting removal of the reaction layer. C-SDP is a synergistic process in which the abrasives remove the passivation film, and the bare surface reacts actively–passively with the polishing slurry to reform the passivation film [24]. In the process of polishing, chemical corrosion of the polishing fluid and mechanical grinding of the abrasives are coupled to achieve material removal at the atomic level and efficient removal of the tungsten surface.

## 3. Experiments

### 3.1. Preparation of Dilatancy Pad and Polishing Slurry

**Figure 2:**
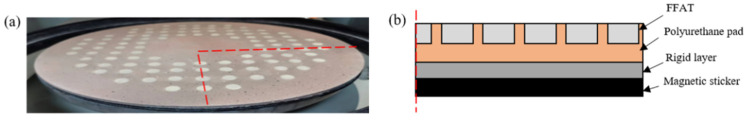
Polyurethane dilatancy pad: (**a**) physical diagram; (**b**) structure diagram.

Viscoelastic materials, ingredients, and abrasives with a certain mass ratio were evenly mixed by mechanical agitation at 80 °C. After that, the mixture was cooled to room temperature to obtain shear dilatancy material suitable for SDP. In our research, the viscoelastic material was a polymer material with non-Newtonian fluid properties, and fumed silica was used as the ingredient to improve the mechanical properties of viscoelastic material. This viscoelastic material is prone to shear hardening under the action of external forces, which can enhance the holding force of abrasives. The dilatancy pad was obtained by filling the shear dilatancy material in a special polyurethane polishing pad, as shown in Figure 2a. The structure of the polyurethane dilatancy pad is shown in Figure 2b. The polyurethane pad filled with the shear dilatancy material is attached to the rigid layer to achieve a certain rigid support effect. The magnetic layer at the bottom makes the dilatancy pad magnetically adsorbed on the surface of the polishing base plate for easy replacement.

The polishing base slurry was prepared by deionized water, the dispersant, and the active agent. Diamond micro-powders with a particle size of 0.5 μm were used as the abrasives. H_2_O_2_ with a concentration of 30 vol.% was used as the oxidant. The pH value of the polishing slurry was adjusted by NaOH. The slurry was continuously stirred for 30 min to ensure that all components were well mixed for the polishing experiments.

### 3.2. Experimental Process and Conditions

**Figure 3:**
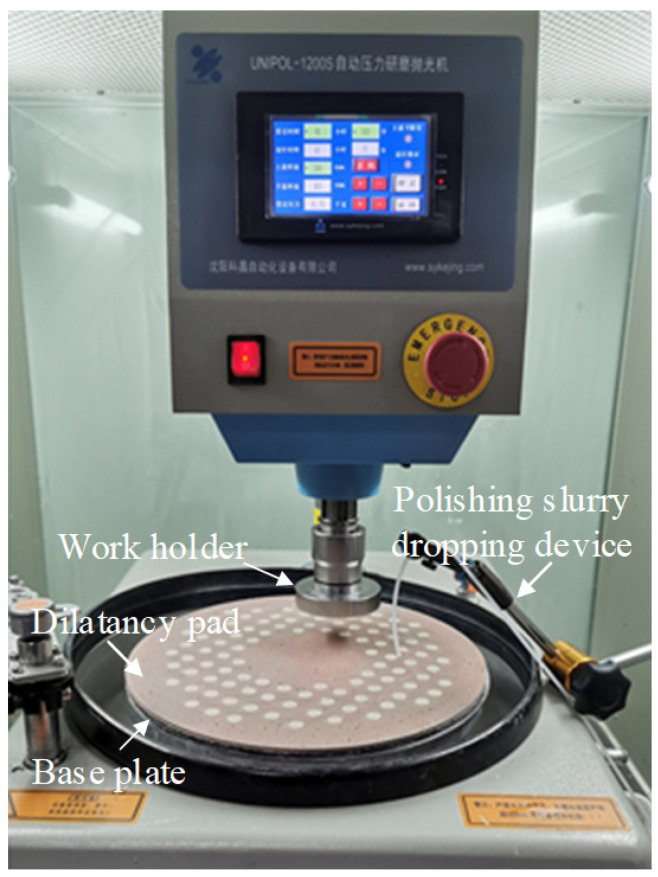
The experimental device of C-SDP.

Tungsten workpieces used for the polishing experiments were obtained by a rolling process, which gave them a high density. The characteristics of tungsten are shown in Table 1. In this study, the plane workpiece was taken as the research target. Tungsten samples were 10 mm in diameter and 0.3 mm in thickness. C-SDP polishing experiments were carried out on the experimental device, as shown in Figure 3. During the polishing process, tungsten samples fixed on the fixture rotated along the normal direction with a certain pressure to ensure a uniform polishing of the workpiece surface.

The experimental conditions are shown in Table 2. In order to achieve better mechanical removal effects of the dilatancy pads, the C-SDP polishing experiments were carried out under the condition of high-pressure and high-speed. Tungsten has a high dissolution rate under alkaline conditions, which can increase the material removal rate. Therefore, the effects of polishing slurries at pH 7, 8, 9, 10, 11, and 12 on the polishing effects of tungsten were studied. Moreover, the effects of H_2_O_2_ concentrations (0–2.0 vol.%) on the polishing performance of tungsten were also investigated.

### 3.3. Measurement and Testing

The pH values of the polishing slurries were measured by a glass electrode pH meter (PB-10, Sartorius, Germany, resolution: 0.01). The *MRR* of tungsten was measured by a precision balance (ME36S, Sartorius, Germany, resolution: 0.001 mg). The formula for calculating *MRR* is as follows:

(1) $$MRR=\frac{\Delta m}{\rho ts}$$

Δ*m* (g) is the quality difference of the tungsten sample before and after polishing, *ρ* (g/cm^3^) is the density of the tungsten sample, *s* (cm^2^) is the area of the tungsten sample, *t* (h) is the polishing time, and the unit of *MRR* is μm/h. Each polishing experiment was repeated three times, and the mean value was calculated.

After polishing, the surface roughness (*R*_a_) of tungsten was measured by a 3D profile White Light Interferometer (Super View W1, Chotest, Shenzhen, China), and the sampling range of the White Light Interferometer was 0.5 × 0.5 mm. The surface morphology of the workpiece was observed by a large-field-depth digital microscope (VHX-7000, Keyence, Osaka, Japan). The dynamic potential polarization curves of tungsten in abrasive-free solutions with different pH values and H_2_O_2_ concentrations were tested by an electrochemical system with a three-electrode cell (CHI760E, CH Instruments, Shanghai, China). The chemical reactions between the components of the polishing slurry and tungsten were analyzed by X-ray photoelectron spectroscopy (ESCALAB 250Xi, Thermo Fisher, Waltham, MA, USA).

## 4. Results and Discussion

### 4.1. Rheological Analysis of Shear Dilatancy Material

Rheological testing of shear dilatancy material was performed by a rotational rheometer (MCR302, Anton Paar, Graz, Austria) at a constant testing temperature of 25 °C. During the test, the distance between the plate clamp and rotor (both 25 mm in diameter) was 1 mm. The strain was constant at 0.1%, and frequency sweep tests were performed from 0.1 to 10 Hz. The measurement for each sample was repeated three times in order to quantify the measurement error.

**Figure 4:**
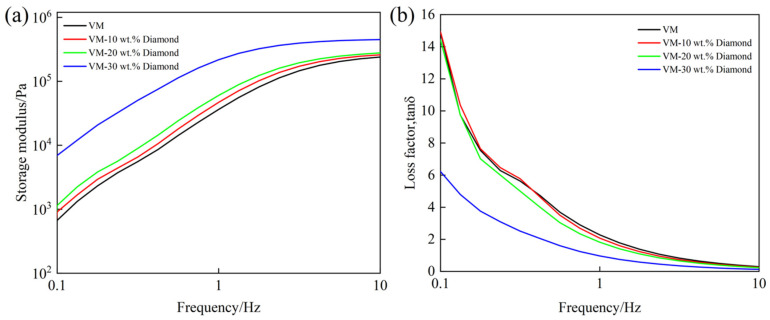
Rheological curves of the shear dilatancy material: (**a**) storage modulus curves; (**b**) loss factor curves.

Figure 4a,b respectively show the trends of *G’* and *tanδ* of samples with different abrasive concentrations as the frequency increased. The *G’* represents the ability of material to store elastic deformation energy, which is used to characterize the elasticity of material. The *tanδ* represents the viscoelastic properties of material. When the *tanδ* is smaller, the elasticity of material is greater. The frequency (*tanδ* = 1) is the critical frequency of the solid–liquid phase transition, beyond which the material transforms from a liquid-like state to a solid-like state. It can be clearly found that with increasing frequency, the *G’* of sample increased, while the *tanδ* decreased. The material experienced an obvious shear hardening effect, which met the requirements of shear dilatancy polishing. As shown in Figure 4a,b, the abrasive concentrations significantly affected the rheological properties of the shear dilatancy material. With the increase of abrasive concentrations, the *G’* of the sample increased, and the *tanδ* decreased. The elasticity of the material was improved, and its phase transition frequency decreased. In other words, the material transition to a “flexible fixed abrasive tool” requires a lower polishing speed, which makes the material more prone to transition from liquid-like to solid-like. When the abrasive concentration was 30 wt.%, the *G’* increased significantly, which was much higher than that of 20 wt.%. Combined with the rheological test results and polishing requirements, VM-30 wt.% Dimond was selected for the preparation of the dilatancy pad.

### 4.2. Effect of pH Values on the Polishing Performance of Tungsten

**Figure 5:**
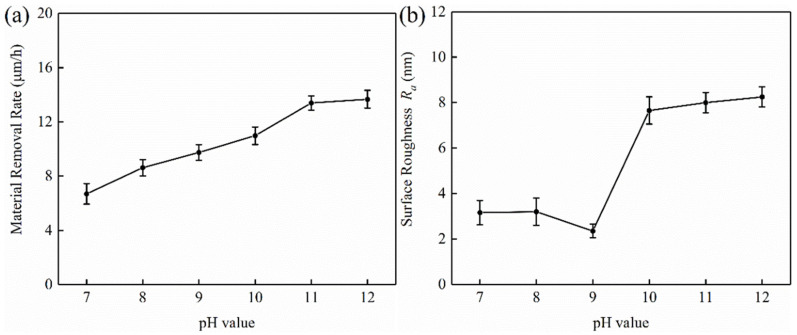
Effect of pH values on the removal rate and surface roughness: (**a**) material remove rate; (**b**) surface roughness.

pH value is an important component of chemical polishing slurry, which determines the basic polishing environment of C-SDP and directly affects the polishing quality [25]. The effects of pH values on tungsten C-SDP are shown in Figure 5. As shown in Figure 5a, with the increase of pH, the material removal rate of tungsten showed a continuous increasing trend, and it increased from 6.69 µm/h to 13.67 µm/h. In neutral and alkaline environments, the oxide formed on tungsten surfaces is unstable and dissolves in solution at a very low rate to form tungstate ions (WO_4_^−^) [26,27], which can remove the oxide on the tungsten surface to a certain extent. On the other hand, since the oxide is softer and easier to remove than tungsten, the corresponding mechanical removal effect is also more pronounced. Because the dissolution rate of this oxide is low, the mechanical action of abrasives will remove most of the generated oxide, thereby exposing a new tungsten surface to continue the chemical reaction. In the neutral and alkaline conditions of the polishing slurry, the removal rate increases with the increase of pH, which is also related to the dissolution rate. The material removal rate includes the mechanical removal of abrasives and the dissolution of oxide.

When pH = 7, the material removal was mainly achieved by the mechanical action of diamond abrasives. Because the chemical action was very small at this time, the corrosion effect on the tungsten surface was extremely weak, resulting in a minimum material removal rate. When pH > 7, it was easy for tungsten to react with the alkaline slurry. Micro-convex peaks on the tungsten surface could be oxidized into relatively soft oxides. Under the mechanical grinding of diamond abrasives and the dissolution of alkaline solution, the generated oxides could be easily removed, and thus the material removal rate was improved.

However, the final tungsten surface quality is determined by the rate of oxide dissolution, the rate of oxide production, and the mechanical action of abrasives. How to control and balance the relationship among the three factors is a key issue that needs to be considered during the polishing process. With the increase of pH values, the surface roughness of the polished tungsten firstly decreased and then increased, as shown in Figure 5b. The surface roughness *R*_a_ was the lowest at pH = 9. As the pH increased from 7 to 9, *R*_a_ decreased from 3.16 nm to 2.35 nm. However, as the pH sequentially increased to 12, *R*_a_ subsequently increased to 8.25 nm.

**Figure 6:**
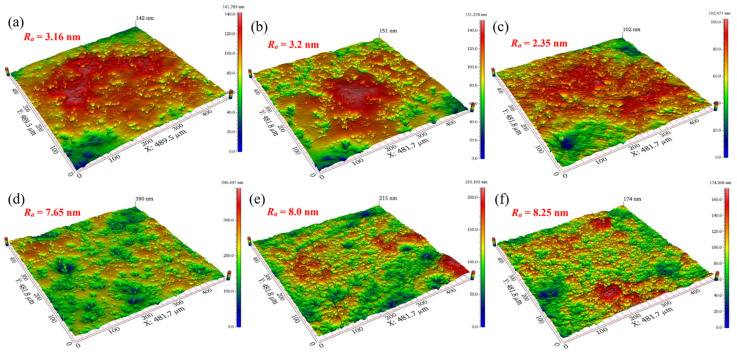
Surface morphologies of polished tungsten under different pH values: (**a**) pH = 7; (**b**) pH = 8; (**c**) pH = 9; (**d**) pH = 10; (**e**) pH = 11; (**f**) pH = 12.

**Figure 7:**
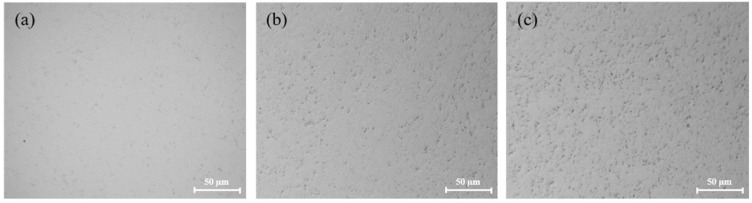
Surface morphologies of polished tungsten under different pH values: (**a**) pH = 9; (**b**) pH = 11; (**c**) pH = 12.

Figure 6 shows the surface morphologies of tungsten after polishing with different pH values. The tungsten surface after polishing was relatively smooth, as seen in Figure 6a–c. However, the tungsten surface became gradually uneven, as seen in Figure 6d–f, and the corrosion degree of tungsten gradually deepened. It can be inferred that when pH > 9, OH^–^ in the slurry was very corrosive for tungsten, which likely caused uneven corrosion of micro-convex peaks on the tungsten surface, resulting in poor surface quality after polishing. Figure 7 shows the surface morphologies of tungsten under different pH values. Under strong alkaline conditions (pH = 11 and pH = 12), the tungsten surface was significantly corroded, as shown in Figure 7b,c.

### 4.3. Effect of H_2_O_2_ Concentrations on the Polishing Performance of Tungsten

**Figure 8:**
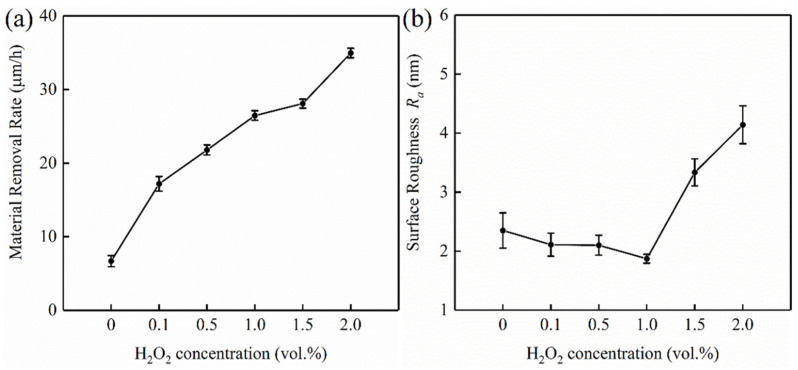
Effect of H_2_O_2_ concentrations on removal rate and surface roughness: (**a**) material removal rate; (**b**) surface roughness.

During the polishing process, a passivation film is formed on the tungsten surface due to oxidizing agents. Because of the low hardness of oxide and the weak interface that exists between tungsten and oxide, the passive film is easily removed by the mechanical action of abrasives [28]. The concentrations of H_2_O_2_ can significantly affect the generation rate of the passivation film on the tungsten surface, which in turn affects the polishing performance of C-SDP [29]. The effects of H_2_O_2_ concentrations on material removal rate and surface roughness of tungsten are shown in Figure 8. In the experiments, the pH values of polishing slurries with different H_2_O_2_ concentrations were all 9. As shown in Figure 8a, with the increase of H_2_O_2_ concentration, the *MRR* of tungsten increased continuously, from 9.71 µm/h to 34.95 µm/h. After the concentration of H_2_O_2_ increased, the chemical corrosion effect of polishing slurry on the tungsten surface material was enhanced, which could generate faster or thicker soft passivation films. As shown in Figure 8b, the surface roughness of tungsten decreased firstly and then increased, and its surface roughness was the lowest when H_2_O_2_ concentration was 1 vol.%. As the H_2_O_2_ concentration increased from 0 to 1 vol.%, *R*_a_ decreased from 2.35 nm to 1.87 nm. *R*_a_ began to increase, reaching 4.14 nm when the H_2_O_2_ concentration was 2 vol.%, since the H_2_O_2_ concentration continued to increase.

**Figure 9:**
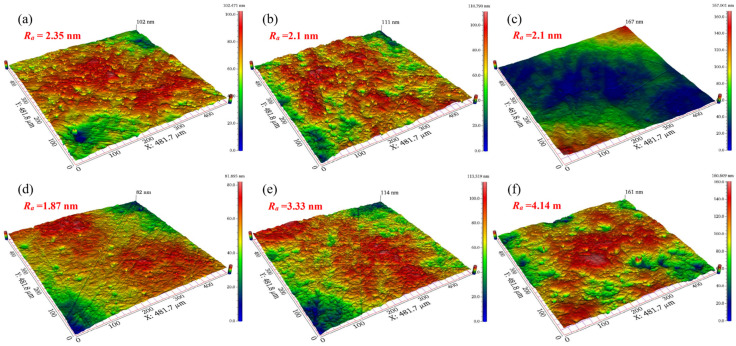
Surface morphologies of polished tungsten under different H_2_O_2_ concentrations: (**a**) 0 vol.%; (**b**) 0.1 vol.%; (**c**) 0.5 vol.%; (**d**) 1.0 vol.%; (**e**) 1.5 vol.%; (**f**) 2.0 vol.%.

Figure 9 shows the surface morphologies of tungsten after polishing with different H_2_O_2_ concentrations. Compared with the different polishing slurries without H_2_O_2_, the tungsten surface became smoother after adding 1 vol.% H_2_O_2_. However, when the concentration of H_2_O_2_ was higher than 1 vol.%, the surface quality of tungsten became slightly deteriorated due to excessive corrosion.

**Figure 10:**
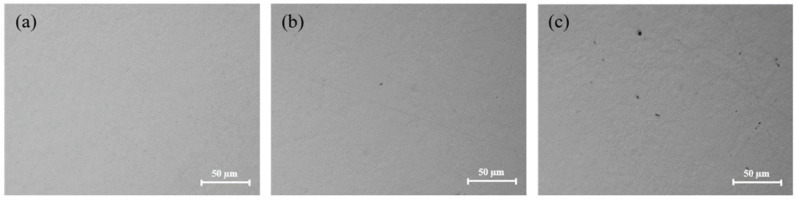
Surface morphologies of polished tungsten under different H_2_O_2_ concentrations: (**a**) 1.0 vol.% H_2_O_2_; (**b**) 1.5 vol.% H_2_O_2_; (**c**) 2.0 vol.% H_2_O_2_.

**Figure 11:**
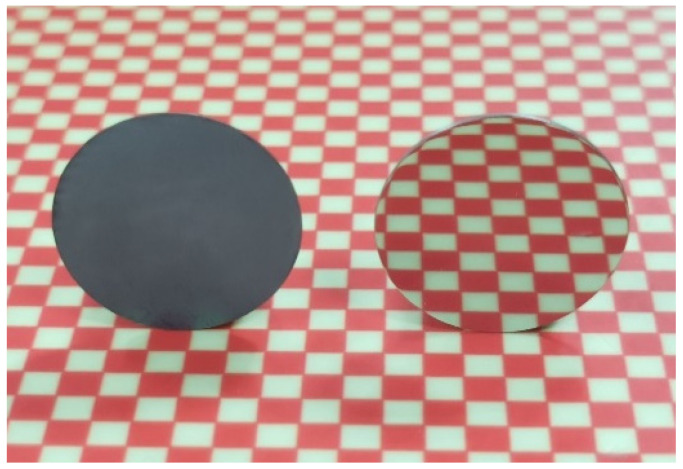
The tungsten samples before and after polishing.

Figure 10 shows the surface morphologies of polished tungsten under different H_2_O_2_ concentrations. As shown in Figure 10c, when the H_2_O_2_ concentration was 2 vol.%, a slight orange peel phenomenon and obvious micropores appeared on the tungsten surface. This phenomenon may be related to the fact that the oxidizing property of polishing slurry was too powerful, so that the mechanical removal of abrasives could not keep up with the formation rate of the passivation film. Moreover, the micropores existing in the tungsten were further enlarged. Tungsten samples before and after polishing are shown in Figure 11. Under the optimum parameters of pH = 9 and 1 vol.% H_2_O_2_ concentration, the surface of the tungsten sample after C-SDP achieved a mirror effect without obvious scratches, pits, or other defects.

### 4.4. Electrochemical Testing Results

**Figure 12:**
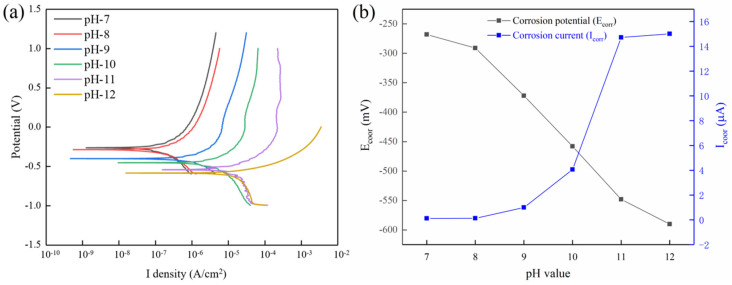
(**a**) Dynamic potential polarization curves at different pH solutions. (**b**) The corrosion potential E_corr_ and the corrosion current density I_corr_ at different pH solutions.

Figure 12a shows the potentiodynamic polarization curves of tungsten in different pH solutions. The composition of electrolyte prepared at different pH values was consistent with that of the polishing slurry except that there was no oxidant. Figure 12b shows the corrosion potential (E_corr_) and the corrosion current density (I_corr_) of tungsten at different pH solutions, which could be obtained from the potentiodynamic polarization curves in Figure 12a.

The more negative the corrosion potential is, the more easily the tungsten surface is corroded [30]. The E_corr_ gradually tended to negative values with increasing pH values, as shown in Figure 12b. Among the six pH values, the E_corr_ was the largest (−268 mV) at pH = 7, which indicated that tungsten was the least susceptible to corrosion at this time. The E_corr_ was the smallest (−590 mV) at pH = 12, and tungsten was most easily corroded at this time. These were consistent with the polishing results shown in Figure 6; the surface quality of tungsten was the worst when pH = 12. In electrochemical experiments, the I_corr_ is used to represent the corrosion rate of the workpiece, which can often reflect the change of *MRR* in the polishing process. As shown in Figure 12b, the I_corr_ increased with increases in the pH values, which meant the corrosion rate of tungsten increased gradually. The maximum I_corr_ was 15.01 μA when pH = 12, which was two orders of magnitude higher than that at pH = 7 (0.1185 μA). This phenomenon corresponded to the maximum *MRR* under the condition of pH = 12 shown in Figure 5a. According to the values of I_corr_ and E_corr_, with increases in pH values, tungsten was more easily corroded, and the corrosion rate increased in the alkaline solution. This phenomenon was consistent with the effect of different pH values on the polishing performance of C-SDP.

**Figure 13:**
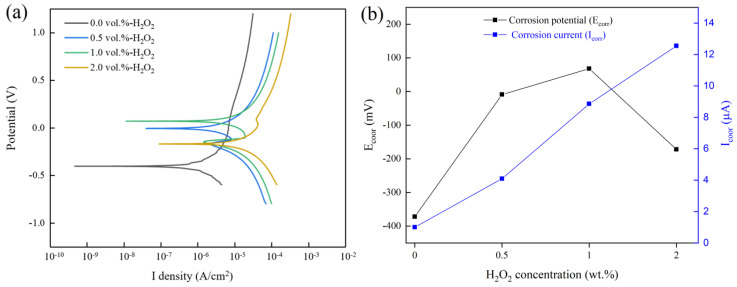
(**a**) Dynamic potential polarization curves at different H_2_O_2_ concentration solutions. (**b**) The corrosion potential E_corr_ and the corrosion current density I_corr_ at different H_2_O_2_ concentration solutions.

Figure 13a shows the potentiodynamic polarization curves of tungsten in different H_2_O_2_ concentration solutions, and four typical H_2_O_2_ concentrations of 0, 0.5 vol.%, 1 vol.%, and 2 vol.% were selected. Except for the different concentrations of H_2_O_2_, the other components of solution were the same as those of the polishing slurry. Figure 13b shows the corrosion potential (E_corr_) and the corrosion current density (I_corr_) of tungsten in different H_2_O_2_ concentration solutions, which could be obtained from the potentiodynamic polarization curves shown in Figure 13a. As shown in Figure 13b, the E_corr_ of tungsten under the H_2_O_2_ concentration of 1 vol.% (68 mV) was higher than that under the H_2_O_2_ concentrations of 0 vol.% (−372 mV), 0.5 vol.% (−9 mV), and 2 vol.% (−172 mV). This indicated that when the H_2_O_2_ concentration was 1 vol.%, the corrosion tendency of the solution for tungsten was the smallest. Between 0 and 1 vol.%, the oxidizing properties of the solution became stronger with the increase of H_2_O_2_ concentration, and the E_corr_ tended to be more positive. It seems that a dense and thick passivation film gradually formed on the tungsten surface, which reduced the corrosion tendency of tungsten. The E_corr_ decreased at 2 vol.% H_2_O_2_. This may be due to the high concentration of 2 vol.% H_2_O_2_, leading to the destruction of the passivation film, which accelerated the dissolution rate of the passivation film in the solution, resulting in a decrease in the corrosion resistance of tungsten. In the four solutions with different H_2_O_2_ concentrations, the I_corr_ increased with increasing H_2_O_2_ concentrations, which were 1.003 µA, 4.094 µA, 8.855 µA, and 12.55 µA, respectively, corresponding to the trend of *MRR* in Figure 8a. When the H_2_O_2_ concentration in solution was 2 vol.%, the I_corr_ was the largest, and it was an order of magnitude higher than that without H_2_O_2_, which indicated that the passivation film was more likely to be formed on the tungsten surface after the addition of H_2_O_2_.

**Figure 14:**
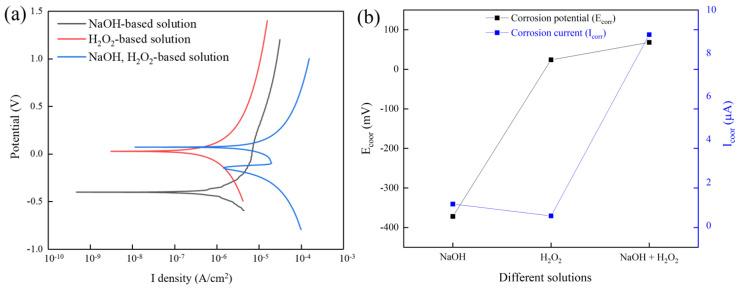
(**a**) Dynamic potential polarization curves at different solutions. (**b**) The corrosion potential E_corr_ and the corrosion current density I_corr_ at different solutions.

Figure 14a shows the potentiodynamic polarization curves of tungsten in solutions with different components. Three solutions with different components were an NaOH-based solution (pH = 9), H_2_O_2_-based solution (1 vol.% H_2_O_2_), NaOH, and H_2_O_2_-based solution (pH = 9, 1 vol.% H_2_O_2_). Figure 14b shows the corrosion potential (E_corr_) and corrosion current density (I_corr_) of tungsten in solutions with different components. As shown in Figure 14b, in the solution without H_2_O_2_ addition, the I_corr_ decreased from 8.855 µA to 1.003 µA, and the E_corr_ changed from 68 V to −372 mV compared with the NaOH and H_2_O_2_-based solutions. This indicated that H_2_O_2_ participated in the chemical reaction and could reduce the corrosion of the tungsten surface. After removing NaOH from the solution, the corrosion current I_corr_ on the tungsten surface was the smallest at only 0.454 µA. The result shows that the combined action of NaOH and H_2_O_2_ can enhance the corrosion rate of tungsten, thereby improving the material removal rate in C-SDP.

It can be seen from the above electrochemical experiments that the chemical corrosion rate and corrosion resistance of tungsten are significantly affected by the chemical agents in the polishing slurry. The etch rate of tungsten can be improved by NaOH and H_2_O_2_ in the polishing slurry, which is consistent with the material removal rate results shown in Figure 5a and Figure 8a. The chemical composition of the tungsten surface in different solutions is analyzed below, and the chemical reactions that occur during the tungsten C-SDP process are discussed.

### 4.5. XPS Testing Results

**Figure 15:**
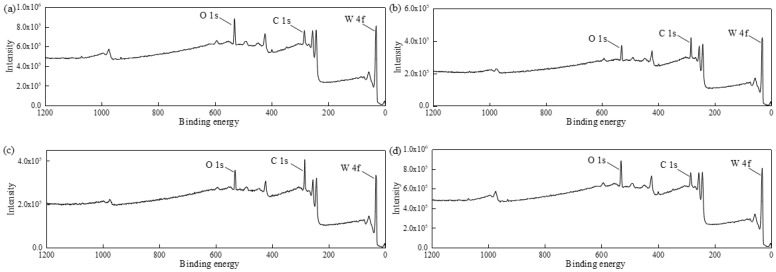
X-ray photoelectron full spectra of the tungsten surface under different conditions: (**a**) pH 9 solution immersion; (**b**) H_2_O_2_ solution immersion; (**c**) polishing slurry immersion; (**d**) after C-SDP.

**Figure 16:**
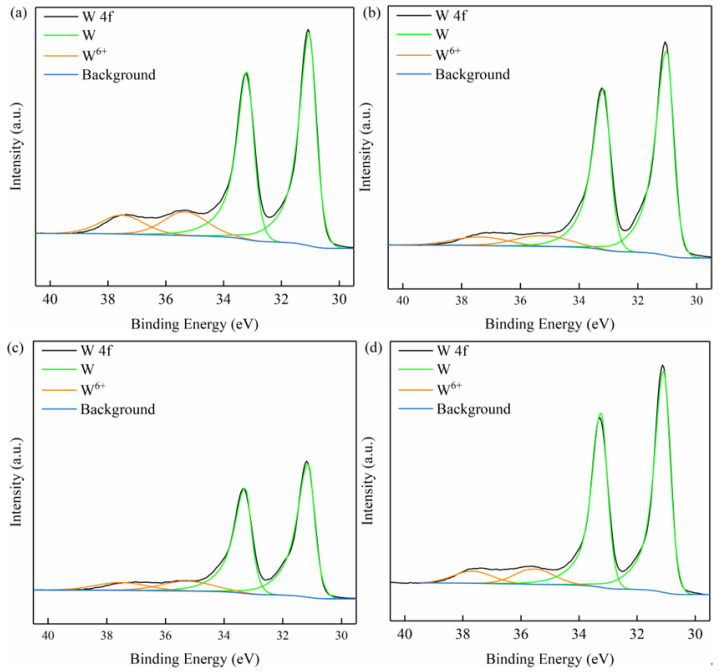
X-ray photoelectron fine spectra of the tungsten surface under different conditions: (**a**) pH 9 solution immersion; (**b**) H_2_O_2_ solution immersion; (**c**) polishing slurry solution immersion; (**d**) after C-SDP.

Figure 15 shows the XPS full spectra of the tungsten surface under different conditions. As seen in Figure 15, the main elements on the surface of tungsten samples were C, O, and W. The C element mainly came from the pollutants adsorbed on the tungsten surface during XPS. The presence of the O element under the four different conditions indicates that oxides were produced on the tungsten surface after soaking or polishing. The XPS fine spectra of the tungsten surface in Figure 16 were further analyzed in terms of the element valence.

Figure 16 shows the XPS fine spectra of the tungsten surface under different conditions. In Figure 16, the deconvolution of the W (4f) spectrum shows four peaks: the two peaks located around 35.5 eV and 37.7 eV can be indexed, respectively, to W 4f_7/2_ and W 4f_5/2_ of W^6+^, while the other two peaks at 31.0 eV and 33.2 eV refer, respectively, to W 4f_7/2_ and W 4f_5/2_ of W [31]. The peaks of the W 4f orbital appeared in pairs, with the peak at the higher binding energy being W 4f_5/2_ and the lower one being W 4f_7/2_.

Anik et al. investigated the anodic behavior of tungsten at different pH values. When the pH < 1, the main dissolution pathway of oxide is H^+^-assisted dissolution. When the pH is between 4 and 6.5, the dissolution of oxide mainly depends on OH^−^-assisted dissolution. Under strong alkalinity (pH > 12.5), the dissolution is achieved by the slow diffusion of OH^–^ to the W surface [32]. Lillard et al. believed that under acidic conditions, the oxides formed on tungsten surface could be divided into an inner barrier layer of WO_3_ and an outer hydrated layer consisting of WO_3_ (H_2_O) [26].

It was found that tungsten underwent chemical reactions in pH 9 solution and H_2_O_2_ solution, and W^6+^ was formed on the tungsten surface, as determined by XPS spectroscopic analysis. Therefore, it is speculated that WO_3_ may exist on the tungsten surface. It is worth noting that the equilibrium information in the Pourbaix diagram shows that WO_3_ can only be formed stably when pH < 4 [26]. However, this diagram reacts the final product at different pH values and does not involve intermediate products. In addition, it was found by XPS characterization that WO_3_ existed in a weakly acidic environment (4.5 < pH < 6.5), and the electrochemical study of tungsten in a weakly alkaline solution (such as pH = 9) showed that the anodic reaction was independent of pH [32]. Weidman et al. believed that the tungsten surface could show limited passivation behavior at neutral and slightly alkaline pH values [33]. Kneer et al. mentioned that a thin protective oxide layer exists on the tungsten surface over almost the entire pH range. When the pH is 4–9, a thin metastable WO_3_ film is formed on the tungsten surface [34]. Generally, the oxide film on the tungsten surface will dissolve rapidly under strong alkaline conditions [33]. Combined with the XPS spectra in Figure 16, it can be inferred that NaOH and H_2_O_2_ react with tungsten, and the main chemical reactions in alkaline C-SDP polishing slurry [35,36] are:

(2) $$W+6{\mathrm{OH}}_{}^{-}\to {\mathrm{WO}}_{3}+3H_{2}O$$

(3) $$W+3H_{2}O_{2}\to {\mathrm{WO}}_{3}+3H_{2}O$$

(4) $${\mathrm{WO}}_{3}+2{\mathrm{OH}}_{}^{-}\to {\mathrm{WO}}_{4}^{2-}+H_{2}O$$

Table 3 analyzes the peak areas of different valence states of tungsten shown in Figure 16. As shown in Table 3, the peak area representing the W element is much larger than that of W^6+^, which indicates that the content of hexavalent compounds on the tungsten surface is very small, so the oxide film is very thin. Compared with the tungsten surface immersed in pH 9 solution, the tungsten surface immersed in the polishing slurry has a higher W element content. This may be due to a relatively dense passivation film formed by the reaction between H_2_O_2_ and tungsten in the polishing slurry, preventing further oxidation of the internal tungsten. On the other hand, the passivation film can react with OH^−^, leading to a reduction in its thickness. The proportion of the W element on the tungsten surface was also increased after C-SDP, which indicates that the addition of mechanical action can reduce the thickness of the passivation film to a certain extent. In general, the hardness of tungsten oxides tends to decrease as the oxidation state increases. Therefore, in the actual C-SDP process, both the dissolution mode and the chemical state of the oxide play a role in the material removal of the tungsten surface. At present, there are few studies on the chemical corrosion mechanisms of tungsten polishing in alkaline environments, and it is necessary to carry out further systematic research in the follow-up.

## 5. Conclusions

In this paper, a novel high-efficiency C-SDP method was proposed to obtain high surface quality tungsten, and the effects of pH values and H_2_O_2_ concentrations on the polishing performance of tungsten were investigated. The experimental results showed that tungsten C-SDP was significantly affected by pH values and H_2_O_2_ concentrations. The *MRR* gradually increased with increasing pH values from 6.69 µm/h to 13.67 µm/h. With increasing H_2_O_2_ concentrations, the *MRR* increased from 9.71 µm/h to 34.95 µm/h. When the pH value was 9 and the H_2_O_2_ concentration was 1 vol.%, the optimal *R*_a_ was 1.87 nm, and the corresponding *MRR* was 26.46 µm/h. This indicates that the C-SDP polishing technique was an effective method to obtain high surface quality tungsten.

The mechanism influences of pH values and H_2_O_2_ concentrations on tungsten C-SDP were clarified by electrochemical and XPS tests. In alkaline polishing slurries containing H_2_O_2_, the tungsten surface mainly undergoes an oxidation reaction to form relatively soft tungsten trioxide, which can be quickly removed by abrasives. Then a new surface of the tungsten workpiece is exposed, and the chemical reaction continues, thereby increasing the material removal rate. In the C-SDP polishing slurry, when the alkalinity is too strong or the concentration of oxidant too high, excessive chemical corrosion and poor surface quality will result.

## Figures and Tables

**Table 1:** Chemical composition and mechanical characteristics of tungsten.

Parameters	Values
Content of W (%)	≥99.99
Density (g/cm^3^)	19.35
Mohs hardness	7.5
Tensile strength (MPa)	980–1078
Fracture toughness (MPa·m^1/2^)	5.4

**Table 2:** Experimental conditions.

Parameter	Values
Top plate polishing speed (r/min)	20
Bottom plate polishing speed (r/min)	120
Polishing pressure (kPa)	468
Abrasive particle size (µm)	0.5
Abrasive concentration of slurry (wt.%)	1
Polishing pad	Polyurethane dilatancy pad
Slurry flow rate (mL/min)	3
pH	7; 8; 9; 10; 11; 12
H_2_O_2_ (vol.%)	0; 0.1; 0.5; 1; 1.5; 2

**Table 3:** Analysis results of XPS peak area of tungsten.

Conditions	W^6+^/W Ratio
pH = 9	0.237
1 vol.% H_2_O_2_	0.137
Slurry	0.181
C-SDP	0.142
